# 1H-Imidazo[4,5-f][1,10]phenanthroline Derivatives as Promising Ligands for Ir and Ru Complex Compounds for Applications in LECs: Mini-Review

**DOI:** 10.3390/ma18184380

**Published:** 2025-09-19

**Authors:** Agnieszka Krawiec, Agata Szłapa-Kula, Sławomir Kula

**Affiliations:** Institute of Chemistry, Faculty of Science and Technology, University of Silesia in Katowice, Szkolna 9 St., 40-007 Katowice, Poland; agnieszka.krawiec@us.edu.pl

**Keywords:** 1H-imidazo[4,5-f][1,10]phenanthroline derivatives, ruthenium complexes, iridium complexes, electroluminescence, light-emitting electrochemical cells, LEC

## Abstract

Light-emitting electrochemical cells (LECs) are attracting significant attention due to their simple design, low production costs, and ability to operate on flexible substrates. As a result, they are increasingly considered a highly attractive alternative to organic light-emitting diodes (OLEDs). The emissive layer is a key element determining the efficiency of LECs. Therefore, considerable attention is currently being paid to finding chemical compounds that could be used as efficient and stable light emitters. Ionic transition metal complexes (iTMCs) are a prime example of such materials. In recent years, iridium and ruthenium complexes containing ligands based on 1H-imidazo[4,5-f][1,10]phenanthroline derivatives have attracted particular interest in LECs. Therefore, this paper discusses in detail the physicochemical properties and application potential of iridium and ruthenium complexes containing these ligands in LECs.

## 1. Introduction

In recent decades, there has been extremely dynamic development of technology, which has affected almost every aspect of everyday life and industry—from modern forms of communication, through diagnostics and therapies in medicine, to the energy sector. This development, although it has opened up enormous opportunities for us, has also led to a sharp increase in demand for electricity. Estimates show that as much as 20% of global electricity consumption is accounted for by lighting [1]. Even a small reduction in electricity demand in this area has a significant, tangible effect on a global scale. Therefore, one of the key challenges posed by this development is the need to create modern, energy-efficient light sources. It has been observed that the use of solid-state lightning (SSL) technology significantly reduces energy consumption [2].

Solid-state lighting (SSL) is a type of lighting that uses semiconductor light-emitting diodes (LEDs), organic light-emitting diodes (OLEDs), or light-emitting electrochemical cells (LECs). SSL devices utilize the phenomenon of electroluminescence (EL), which is the emission of light caused by the action of an electric field or the flow of electric current. The material responsible for generating this light is called a phosphor [3]. Unlike traditional lamps, SSL devices are characterized by higher luminous efficacy [4]. The use of SSL helps reduce energy losses, which in older technologies mainly result from heat emission instead of light. In addition, modern SSL solutions offer extra advantages, such as the ability to adjust the color and intensity of light, longer lifespan, and greater flexibility in designing contemporary lighting forms [4].

LED technology is based on inorganic semiconductors composed of various elements such as indium (In), gallium (Ga), phosphorus (P), and nitrogen (N). Thanks to this, it is possible to achieve highly efficient point light sources in a wide range of colors. Another advantage of such semiconductors is their high resistance to mechanical damage, which is why LEDs are now widely used, among other applications, in screen backlighting, traffic signaling, and general lighting [5].

In recent years, OLED technology has gained significant popularity among researchers as well as in the consumer market. OLED technology stands out for its many advantages, one of the key ones being the lack of need for a separate backlighting system. Thanks to this, it is possible to design exceptionally thin displays [6,7,8]. Moreover, OLED technology allows for precise dimming of selected screen areas, which makes it possible to achieve deep, perfect blacks. As a result, displaying dark content is associated with a significant reduction in energy consumption. OLED displays are also characterized by high contrast, a wide color gamut, and very fast response times, which makes them especially valued in applications related to gaming and computer graphics [8].

**Scheme 1:**
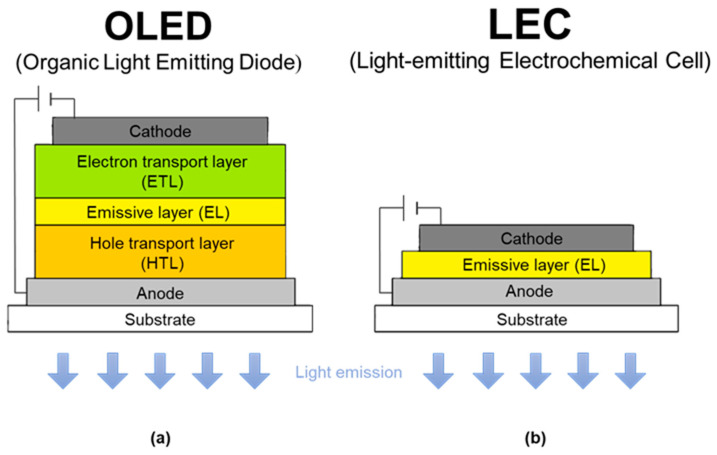
Simplified structure of an OLED (**a**) and an LEC (**b**) [13,24,25,26].

In a simplified diagram, OLEDs consist of a substrate, a cathode, an anode, hole transport layers (HTL), electron transport layers (ETL), and an emissive layer (EML) (Scheme 1a) [9,10]. As mentioned earlier, the operation principle of the diode is based on the phenomenon of electroluminescence. When voltage is applied to the electrodes, electrons injected from the cathode and holes injected from the anode move from the conducting layers to the emissive layer. In this layer, due to electrostatic interactions, electrons and holes approach each other and recombine. As a result, electron–hole pairs (excitons) are formed, which are responsible for generating light [4,7].

An LEC operates on a similar principle. LECs consist of a layer of ionic luminescent material located between two electrodes (Scheme 1b) [11,12,13,14,15]. Under the influence of an applied voltage, ions migrate toward the electrodes and accumulate at the interface, where the metallic layer physically blocks their further movement. As a result, the accumulation of ions at the electrode interfaces reduces the energy barrier necessary for p- and n-type doping in the emissive layer [16,17,18,19]. An important difference is the possibility of using electrodes resistant to exposure to air (such as Al, Au, and Ag), which means that the production process does not require vacuum conditions—a necessity in the case of OLEDs. There is also no need for strict encapsulation of selected elements and layers. This simplified structure and the less restrictive production conditions translate into lower manufacturing costs [17,20]. Although LECs have many advantages, they do suffer from certain limitations, such as a short operational response time and consequently a long turn-on time (ton—the time needed to reach maximum brightness) [21,22,23,24,25,26]. Furthermore, the state of knowledge regarding efficient and stable emitters that provide favorable LEC parameters is still limited. To date, various types of ionic emitter materials, such as conjugated polymers (CPs) [13], organic small molecules (SMs) [20,27], quantum dots [28], perovskite nanoparticles (NPs) [29], and ionic transition metal complexes (iTMCs) [13,30], have been successfully used in LECs. In this review, special attention is devoted to iTMC compounds, which represent one of the most promising classes of emitters for use in LECs.

iTMC compounds have already been studied as agents used in biological imaging [31] and in photodynamic cancer therapy [32], as well as emitters in OLEDs [33]. They offer many advantages in the context of optoelectronic applications. Since the principle of operation of LECs requires the emissive layer to be ionic in nature, iTMCs—unlike polymers—are inherently ionic. Therefore, it is not necessary to additionally introduce ionic composites into the emissive layer. Moreover, the use of small anions (e.g., BF_4_^−^, ClO_4_^−^) increases ionic conductivity, which shortens the device turn-on time (t_on_) [23,34]. iTMCs are soluble in polar solvents, making them easy to use in the fabrication of solution-processable devices (e.g., spin coating) [21,23]. The phosphorescent nature of iTMCs makes it possible to utilize 100% of the generated excitons (both singlet and triplet), resulting in significantly higher electroluminescence (EL) efficiency compared to fluorescent materials (such as conjugated polymers or organic small molecules), which use only singlet excitons (~25%). These compounds also exhibit stable electrochemical (red/ox) properties, which supports device durability and operational consistency [13,35].

**Scheme 2:**
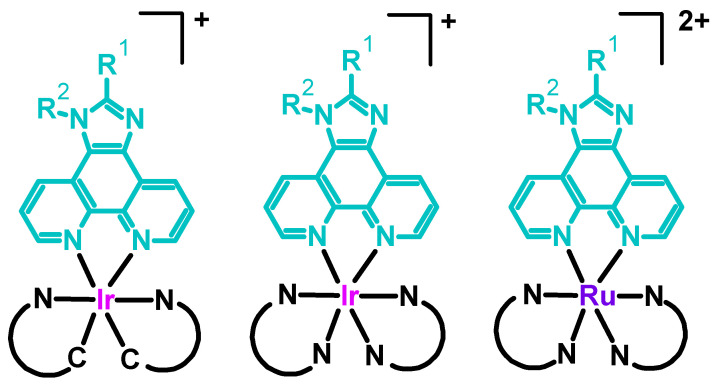
General structures of the analyzed ruthenium and iridium complexes.

The most commonly used metal ions in these complexes are ruthenium Ru(II) and iridium Ir(III), due to their efficient photoluminescent properties and high stability [5]. Iridium complexes investigated for application in LEC devices are generally based on a cyclometalating C^N-type ligand, such as 2-phenylpyridine (ppy) (Scheme 2). They are usually accompanied by an ancillary N^N-type ligand, which can include bipyridine (bpy) or 1,10-phenanthroline (phen) derivatives. In contrast, ruthenium complexes considered for use in LEC devices are dominated by N^N-type ligands (Scheme 2). It is important to emphasize that the choice of ligands for synthesizing the final coordination complexes is never accidental. This is because the properties of the final complex can be widely tuned to specific requirements through careful design and modification of the selected ligands. This allows control over emission color, quantum efficiency, and material stability [36]. One interesting and increasingly studied example includes ligands based on derivatives of 1H-imidazo[4,5-f][1,10]phenanthroline. This group of compounds stands out for its several favorable features, such as the ability to form strong bonds with transition metals, a rigid and planar structure ensuring high thermal stability, and a π-conjugated system that promotes efficient charge transport and high fluorescence efficiency. Additionally, the physicochemical properties of 1H-imidazo[4,5-f][1,10]phenanthroline derivatives can be easily modified by changing substituents at the N1 and C2 positions of the imidazole ring [37,38]. This makes it possible to influence the energies of the frontier orbitals (HOMO and LUMO) and molecular polarity, as well as the color and luminescence efficiency of the complex. Such flexibility in ligand design means that transition metal complexes incorporating 1H-imidazo[4,5-f][1,10]phenanthroline derivatives represent a promising class of materials for applications in modern electroluminescent devices [5,19,23,30,36,37,38,39,40,41,42,43,44,45].

The aim of this literature review was to discuss the influence of ligands from the group of 1H-imidazo[4,5-f][1,10]phenanthroline derivatives on the physicochemical properties and potential applications of iridium and ruthenium coordination complexes in LEC devices (Scheme 2). This work provides a detailed analysis of the research results described so far regarding the synthesis, physicochemical properties, and application possibilities of these complexes. This review may prove extremely useful in designing new emitters for light-emitting electrochemical cells (LECs).

## 2. Synthesis

**Scheme 3:**
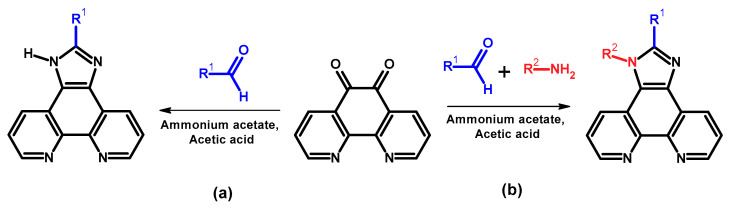
Synthesis of 1H-imidazo[4,5-f][1,10]phenanthroline derivatives, (**a**) without amine and (**b**) with amine [5,19,23,30,36,37,38,39,40,41,42,43,44,45,46,47,48,49,50,51,52,53,54,55,56].

1H-imidazo[4,5-f][1,10]phenanthroline derivatives as ligands have been of interest in the scientific literature for many years [5,19,23,30,36,37,38,39,40,41,42,43,44,45,46,47,48,49,50,51,52,53,54,55,56]. From a synthesis perspective, their attractiveness stems primarily from the central imidazole ring. In the case of the ligands under consideration, this heterocyclic ring is formed during a condensation reaction. Thanks to this, substituents at the N1 and C2 positions can be introduced into the final ligand structure through the appropriate selection of substrates [5,19,23,30,36,37,38,39,40,41,42,43,44,45,46,47,48,49,50,51,52,53,54,55,56]. These substituents can be aliphatic, aromatic, or heteroaromatic. This enables the design and synthesis of a vast range of compounds with properties tailored to specific applications. Their simple synthesis is another advantage of 1H-imidazo[4,5-f][1,10]phenanthroline derivatives [5,19,23,30,36,37,38,39,40,41,42,43,44,45,46,47,48,49,50,51,52,53,54,55,56]. It involves the condensation of 1,10-phenanthroline-5,6-dione, ammonium acetate, and a selected aldehyde (Scheme 3a). Through use of the aldehyde, a substituent is introduced into the ligand structure at the C2 position. A dialdehyde or trialdehyde can also be used in the reaction. This allows for preparation of derivatives containing more than one 1H-imidazo[4,5-f][1,10]phenanthroline core connected via a di- or trialdehyde-derived substituent. Adding an aromatic or heteroaromatic amine during the condensation allows for introduction of a substituent at the N1 position (Scheme 3b). As with the aldehyde, using a diamine allows for synthesis of ligands containing two 1H-imidazo[4,5-f][1,10]phenanthroline cores connected via a diamine-derived substituent. Acetic acid is the most commonly used reaction medium during the synthesis of the ligands discussed [5,19,23,30,36,37,38,39,40,41,42,43,44,45,46,47,48,49,50,51,52,53,54,55,56]. In the case of reaction conditions, the reaction mixtures are heated to boiling temperature, most often for 24 h [19,23,37,39,40,41,43,45]. The synthesis time is shortened [36] or extended [42,44] in selected examples described in the literature, but this is most likely related to the substrates used. An inert gas atmosphere (usually nitrogen) is often used during synthesis [19,23,37,39,40,41,42,44,45]. After the reaction, the mixture is neutralized by adding water and aqueous ammonia. When precipitates form, the crude products are isolated from the reaction medium by extraction or filtration. Crystallization is commonly used to purify 1H-imidazo[4,5-f][1,10]phenanthroline derivatives. In selected cases, column chromatography has also been used, although this is a less common purification method for this group of compounds.

**Scheme 4:**
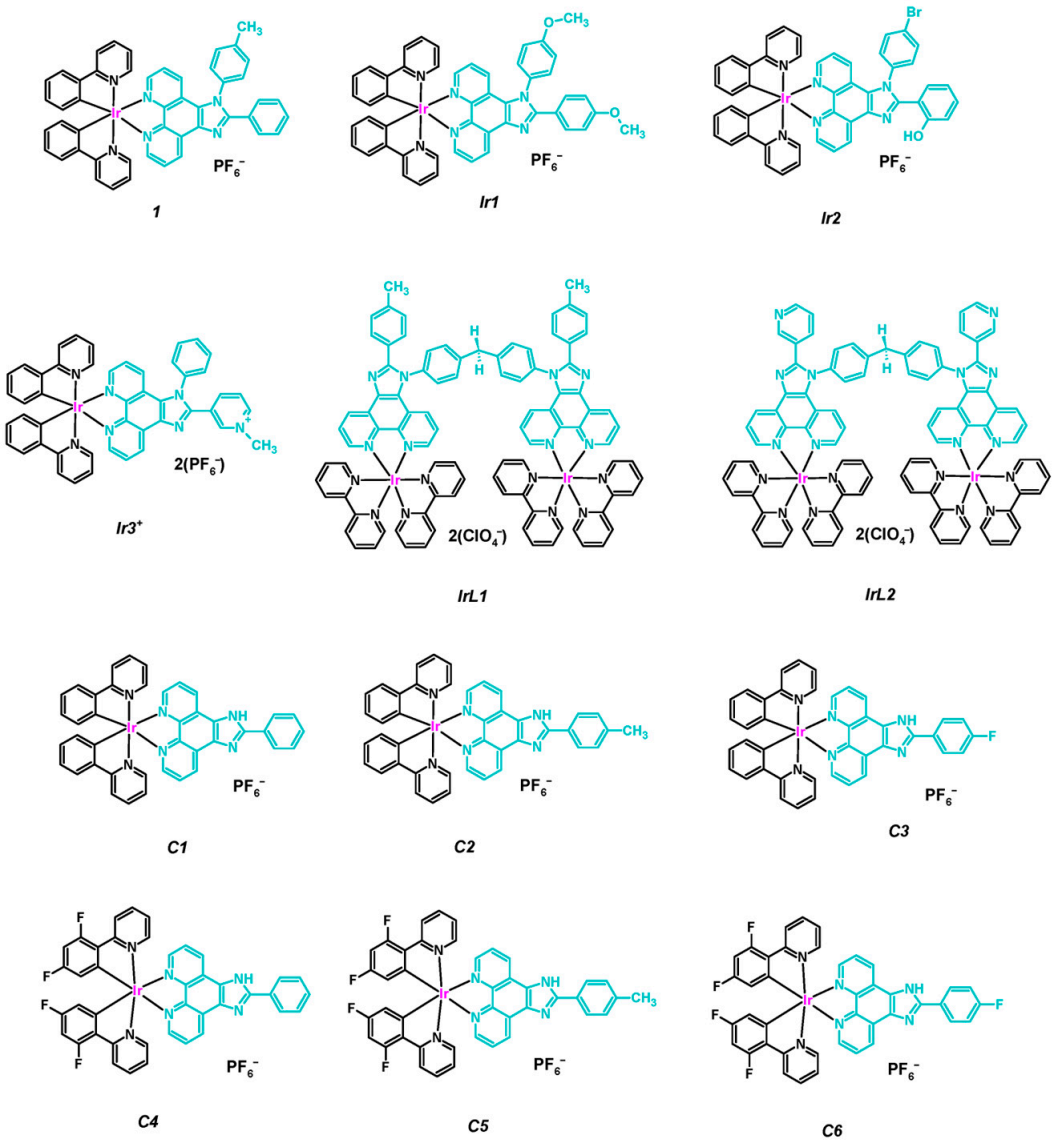
Iridium(III) complexes investigated for use as emitters in light-emitting electrochemical cells (LECs) [30,36,38,43].

All iridium(III) complexes investigated for use as emitters in light-emitting electrochemical cells were obtained by reacting the appropriate bimetallic precursor with the selected derivative 1H-imidazo[4,5-f][1,10]phenanthroline [30,36,38,43]. The structures of compounds described in the literature are shown in Scheme 4.

**Scheme 5:**
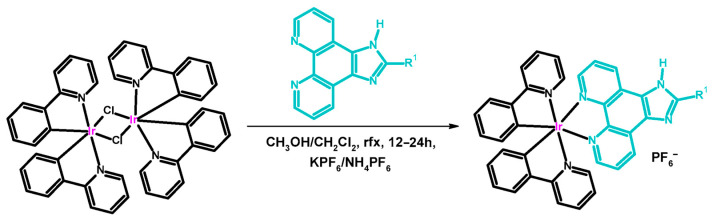
Synthesis of iridium(III) complexes containing one metal center [30,36,43].

Complexes containing one metal center were synthesized by the reaction of dichlorotetrakis(2-(2-pyridinyl)phenyl)diiridium(III) [(ppy)_2_IrCl]_2_ or dichlorotetrakis(2-(2-pyridinyl)-4,6-difluorophenyl)diiridium(III) [(ppy-F_2_)_2_IrCl]_2_ with the appropriate derivative 1H-imidazo[4,5-f][1,10]phenanthroline (Scheme 5) [30,36,43]. All syntheses were performed using a mixture of dichloromethane and methanol as solvents. The reaction mixtures were heated to reflux for 12 to 24 h under an inert atmosphere [30,36,43]. PF_6_^−^ counterions were introduced into the reaction mixtures with the addition of potassium hexafluorophosphate (KPF_6_) [36,43] or ammonium hexafluorophosphate (NH_4_PF_6_) [30]. The synthesized complexes were purified by column chromatography, crystallization, and reprecipitation [36,43]. Thanks to the applied procedure, compounds **1**, **Ir1**–**Ir3**, and **C1**–**C6** were obtained (Scheme 4).

**Scheme 6:**
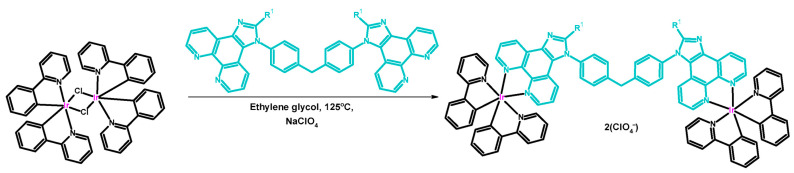
Synthesis of dinuclear iridium(III) complexes [38].

Dinuclear iridium(III) complexes were obtained by reaction of dichlorotetrakis(2-(2-pyridinyl)phenyl)diiridium(III) [(ppy)_2_IrCl]_2_ with ligands having two 1H-imidazo[4,5-f][1,10]phenanthroline fragments (connected together via a substituent at the N1 position) (Scheme 6) [38]. Ethylene glycol was used as the reaction medium. Syntheses were carried out at 150 °C for 18 h under an inert gas (nitrogen) atmosphere. The ClO_4_^−^ counterion was introduced into the reaction mixtures by adding a saturated aqueous sodium perchlorate (NaClO_4_) solution [38]. The obtained **IrL1** and **IrL2** complexes were purified by column chromatography.

**Scheme 7:**
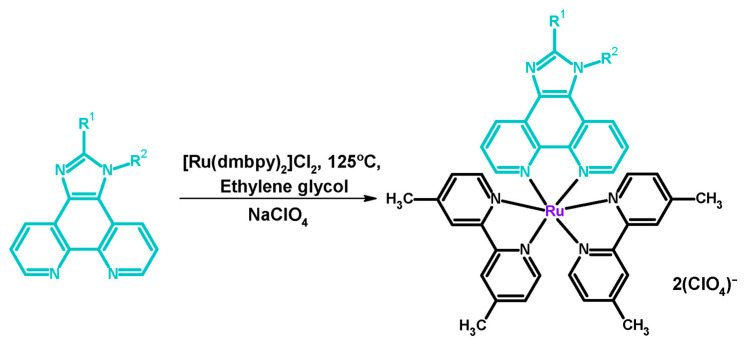
Example of synthesis of a single-core ruthenium(II) complex [23].

**Scheme 8:**
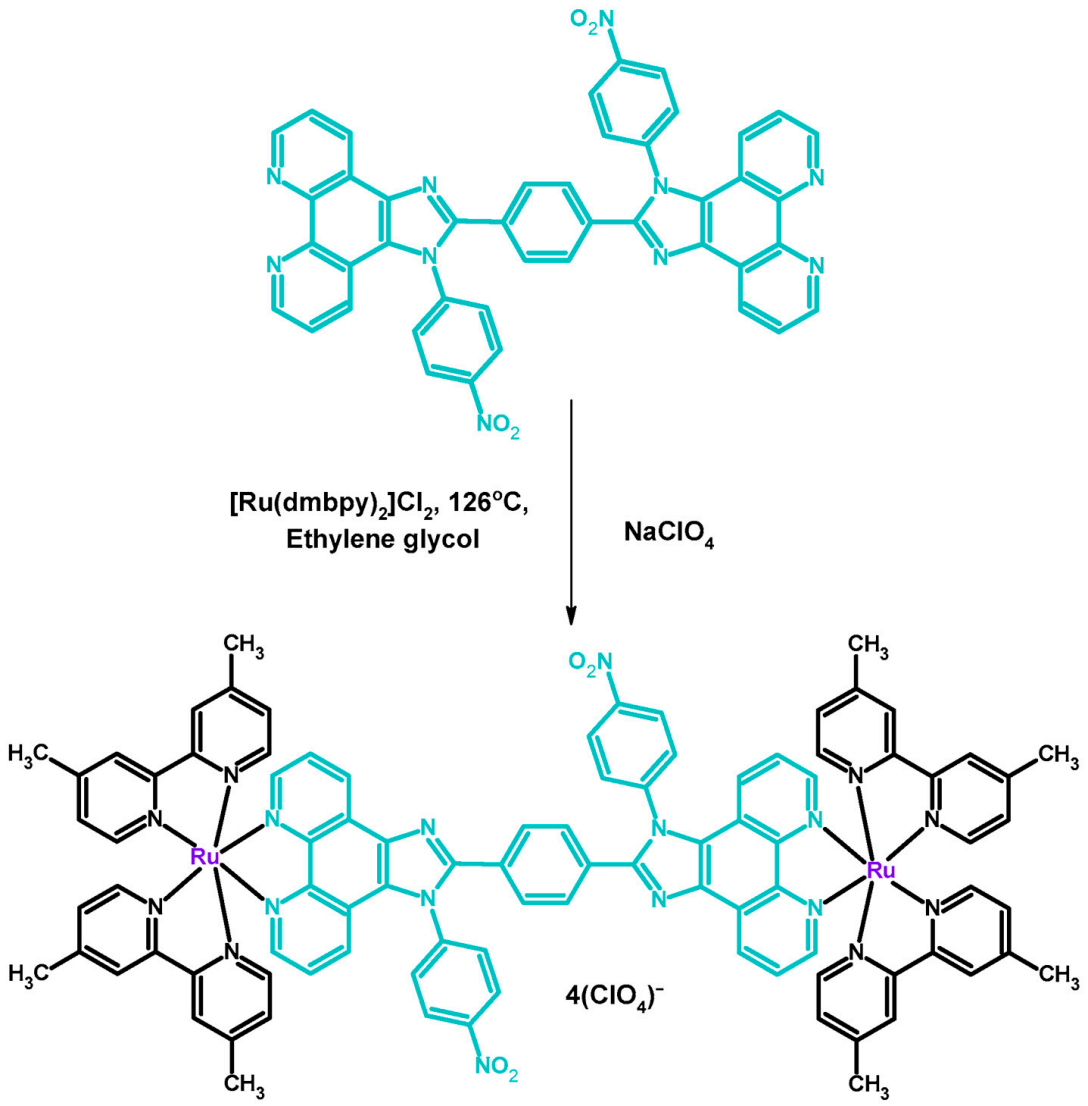
Example of synthesis of a dinuclear ruthenium(II) complex [40].

**Scheme 9:**
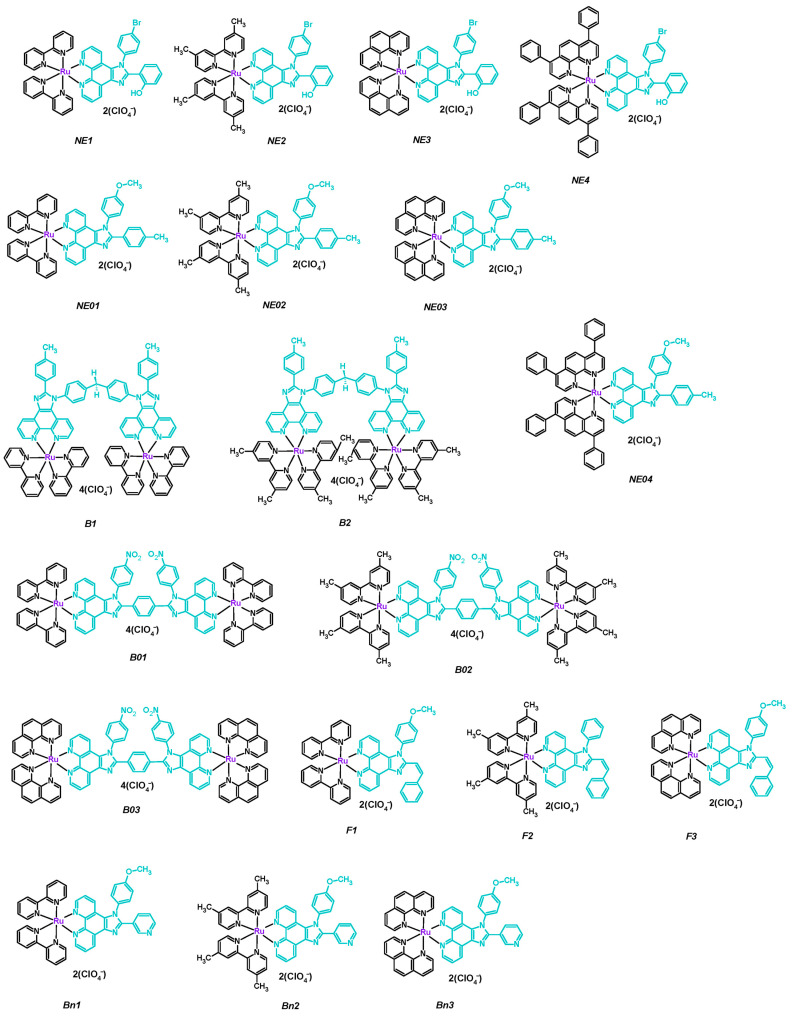

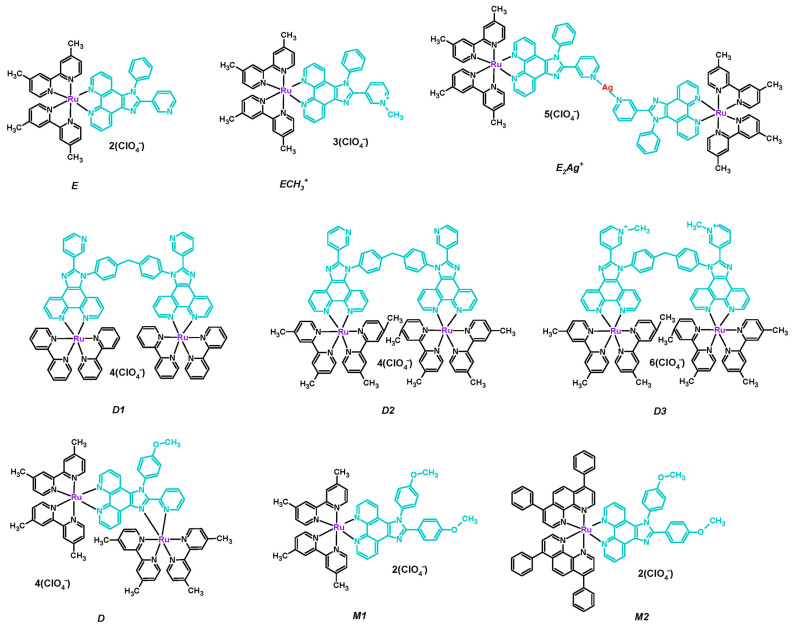
Ruthenium(II) complexes investigated for use as emitters in light-emitting electrochemical cells (LECs) [19,23,37,39,40,41,42,44,45].

All single-core and dual-core ruthenium(II) complexes tested for use as emitters in LECs were synthesized by reacting the metal precursor with 1H-imidazo[4,5-f][1,10]phenanthroline derivatives (Scheme 7 and Scheme 8) [19,23,37,39,40,41,42,44,45]. The precursor used was cis-[Ru(N^N)_2_Cl_2_], in which N^N denotes the following: bpy—2,2′-bipyridine, dmbpy—4,4′-dimethyl-2,2′-bipyridine, phen—1,10-phenanthroline, and bathophen—4,7-diphenyl-1,10-phenanthroline. The structures of ruthenium(II) complexes described in the literature are shown in Scheme 9. The reaction medium used was ethylene glycol [19,23,37,39,40,41,42,44,45] or, in selected cases, acetonitrile [37]. The reaction mixtures were heated at 125–130 °C for 12–24 h using an inert gas atmosphere [19,23,37,39,40,41,42,44,45]. Similarly to the case of dinuclear iridium(III) complexes, the ClO_4_^−^ ion was introduced into the reaction mixtures by adding a saturated aqueous solution of sodium perchlorate (NaClO_4_) [19,23,37,39,40,41,42,44,45]. Column chromatography was the most commonly used method for purifying ruthenium(II) complexes. The following compounds were obtained using this method: **NE1**–**NE4**, **NE01**–**NE04**, **B1**–**B2**, **B01**–**B03**, **F1**–**F3**, **Bn1**–**Bn3**, **E**, **ECH_3_^+^**, **E_2_Ag^+^**, **D1**–**D3**, **D**, and **M1**–**M2**.

## 3. Electrochemical Properties

Detailed electrochemical studies enable us to obtain knowledge about the electrochemical gap and redox processes occurring in the compounds under study. This allows us to determine the HOMO and LUMO values easily. These parameters are fundamental in the design of various devices utilizing chemical compounds. In LECs based on metal complexes, electron and hole transport occur through successive oxidation and reduction of the metal complex during device operation. Therefore, to properly understand the operation of LECs, it is necessary to understand the redox behavior of ionic transition metal complexes (iTMCs) [30]. Considering the above, we would like to discuss the redox properties of iridium and ruthenium complexes containing 1H-imidazo[4,5-f][1,10]phenanthroline derivatives in the complex structure.

Electrochemical studies of all presented complexes were carried out using cyclic voltammetry (CV) [19,23,30,36,37,38,39,40,41,42,43,44,45] and differential pulse voltammetry (DPV) [30]. Measurements were mainly performed in acetonitrile (MeCN) with 0.1 M tetrabutylammonium perchlorate (TBAP) [19,23,30,37,39,40,41,42,44,45] or tetrabutylammonium hexafluorophosphate ((Bu_4_N)PF_6_) [38,43] as the electrolyte. For electrochemical tests, a three-electrode system (Pt disk or glassy carbon, Pt wire, and Ag/AgCl as the working electrode, counter electrode, and reference electrode) and inert gas conditions were used [19,23,30,36,37,38,39,40,41,42,43,44,45]. The obtained data are presented in Table 1.

The first compound with an imp ligand (a derivative of 1H-imidazo[4,5-f][1,10]phenanthroline) studied electrochemically in the context of LEC devices was the iridium(III) complex (**1**) [43]. In 2012, D. Tordera et al. performed electrochemical tests on this complex, determining its oxidation and reduction potentials [43]. Based on these results, they also calculated the electrochemical gap. They observed that this complex undergoes reversible reduction at the imp fragment and quasi-reversible oxidation of the Ir-phenyl fragment. Furthermore, they used dimethylformamide (DMF) as the measurement medium [43]. The results in MeCN and DMF were similar (Table 1). In 2023, B. Bideh et al. published the results of studies on **Ir1**, **Ir2**, and **Ir3^+^** [30]. These complexes differed only in the auxiliary imp ligand. The study revealed that these compounds exhibited a reversible oxidation peak in the positive potential region. This process results from Ir(III) oxidation to Ir(IV) with a substantial contribution from the cyclometalated ligand, phenyl pyridine. All complexes also exhibited a reversible or quasi-reversible reduction process. The authors concluded that this corresponds to a process occurring on the auxiliary ligand, i.e., the 1H-imidazo[4,5-f][1,10]phenanthroline derivative, with a minor contribution from the Ir(III) center [30]. It is worth emphasizing that these complexes exhibit good electrochemical stability due to the reversibility of redox processes. This will result in stable iTMC-LECs being obtained. In analyzing the effect of the complexes’ structure on electrochemical properties, it can be observed that the oxidation potential of all compounds is nearly constant (Table 1). This indicates that the auxiliary ligand does not affect the HOMO orbital value. The situation is different when considering the reduction process. The reduction potential of **Ir1** (−1.81 V) is significantly cathodically shifted (about 0.2 V) relative to **Ir2** (−1.61 V). This means that **Ir2** destabilizes the LUMO orbital more. The presence of peripheral electron-donating (OCH_3_) and -withdrawing groups (Br) affects the reduction potential of the PI ligands that destabilize and stabilize the LUMO levels of cyclometalated complexes, respectively [30]. The authors supported these conclusions with DFT calculations that confirmed their assumptions [30]. In 2024, B. Vásquez et al. studied six new iridium complexes [36]. In their work, they demonstrated that during the oxidation process, quasi-reversible results attributed to Ir(III)/(IV) oxidation could be successfully obtained for the **C1**–**C3** complexes. Interestingly, the **C4**–**C6** compounds exhibited irreversible behavior in this process. Additionally, more-positive oxidation energy values were obtained in the **C4**–**C6** series than for **C1**–**C3**. This state of affairs is related to the higher electron-accepting nature of the F_2_ppy ligands. The authors also noted that the reduction process occurs within a very narrow range for all the presented complexes. They determined that this is related to the weak electronic influence exerted on the 1H-imidazo[4,5-f][1,10]phenanthroline ligand by the substituents at the R2 position. Considering the binuclear imp derivatives, we also obtain the same observations [38]. In cathodic scanning, there is one quasi-reversible or reversible reduction wave associated with the 1H-imidazo[4,5-f][1,10]phenanthroline ligands, as described by M. Mazaheri et al. [38]. The authors associated the small difference in the reduction potential values between **IrL1** and **IrL2** with the difference in the energy gap values of the complexes and their maximum emission. They also showed that this small difference causes a change in the complexes’ LUMO energy, and the difference in maximum emission is caused by the substitution of the group at the C2 position of the 1H-imidazo[4,5-f][1,10]phenanthroline fragments.

**Figure 1:**
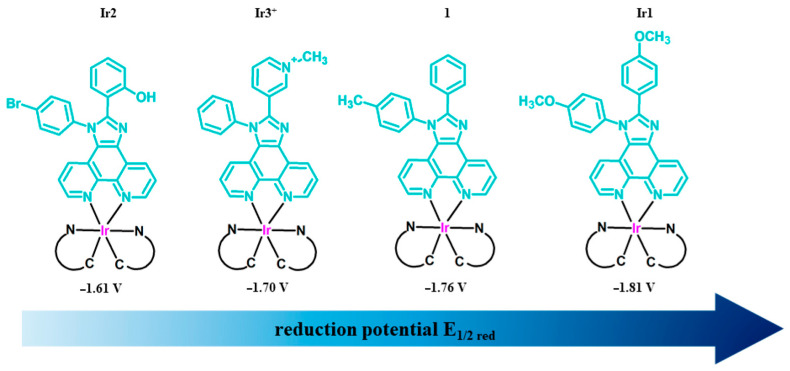
The influence of the substituent in the 1H-imidazo[4,5-f][1,10]phenanthroline derivative on the reduction potential.

Considering all the complexes with the imp ligand studied so far in the context of LECs, an interesting relationship can be observed. As the functional group characteristics in the substituent at the C2 or N1 position of the 1H-imidazo[4,5-f][1,10]phenanthroline derivative change, the reduction potential shifts cathodically (Figure 1). Consequently, the LUMO orbital is more stabilized. We also observe an increase in the electrochemical gap (2.52 eV **Ir2** > 2.59 eV **Ir3^+^** > 2.64 eV **1** > 2.68 eV **Ir1**).

1H-imidazo[4,5-f][1,10]phenanthroline derivatives have also been used to synthesize ruthenium(II) complexes. In 2016, B. Bideh et al. tested four cationic ruthenium(II) complexes [45]. All of them exhibited a multistep oxidation process concentrated on the metal and a multistep reduction focused on the ligand. Interestingly, **NE1** and **NE2** exhibited reversible behavior in the positive potential region, while **NE3** and **NE4** exhibited quasi-reversible waves. This process is attributed to the Ru(II)/Ru(III) redox couple. The authors compared **NE1** to [Ru(bpy)_3_]^2+^, demonstrating a significant anodic shift in the reduction value of the compound relative to the standard (−1.02 V for **NE1** and −1.31 V for the standard). This fact highlights the better π-electron acceptor characteristics of the ligand (1H-imidazo[4,5-f][1,10]phenanthroline derivative) contained in the **NE1** complex. Furthermore, the presence of imp reduces the electrochemical gap of **NE1**. In the same year, the **NE01**–**NE04** complexes were also described [19]. They differed from their predecessors only in the ligand imp. As with their predecessors, they exhibited a reversible Ru(II)/Ru(III) process at positive potentials. At negative potentials, irreversible reductions were observed on the ligands. Studies have shown that **NE02** has the lowest positive oxidizing potential, indicating the greatest destabilization of the HOMO orbital in this compound. In 2017, B. Bideh and H. Shahroosvand conducted research on dinuclear ruthenium derivatives (**B1** and **B2**) [42]. Their voltammograms showed oxidation and reduction peaks corresponding to the Ru(II) → Ru(III) and Ox/Red transitions of the ligands. These studies revealed that the second oxidation peak was caused by the 1H-imidazo[4,5-f][1,10]phenanthroline ligand. Furthermore, in analyzing the results presented by the authors, it can be concluded that complex **B2** has a more destabilized HOMO orbital than complex **B1**. This is due to methyl groups on bipyridine, as shown by DFT calculations [42]. The results for complexes **B01**, **B02**, and **B03** were obtained in a continuation of the conducted research [40]. The compounds under consideration were also dinuclear ruthenium derivatives, but differed in the way the imp ligand linked them. A reversible oxidation process was observed for them, which was attributed to the Ru(II)/Ru(III) transition. Interestingly, the value of this potential depended on the auxiliary ligand used (Table 1). Peaks were also observed in the negative potential region, which could be attributed to processes occurring on the ligands. Moreover, changing the imp ligand in the **B02** complex decreased the oxidation potential by 0.06 V relative to **B2** [40]. As is known, the key factor for LECs is the reversibility of the redox reaction in the ruthenium complexes studied. The complexes (**F1**–**F3**) described in 2019 meet this condition [39]. Studies have shown that they exhibit reversible behavior in the positive potential region associated with Ru(II)/Ru(III). The negative potential in these molecules is attributed to the ligands. The lowest positive potential is observed for **F2**. The electron-donating dimethyl group in the auxiliary ligand causes this. B. Bideh et al. also showed a linear correlation between the square root of the scan rate and the anode current. They showed that the kinetics of the process is controlled by mass transport. The electron transfer rate is, at all potentials, greater than the mass transport rate, and the peak potential is independent of the applied voltage scan rate [39]. In 2020, the **Bn1**–**Bn3** complexes were also described [41]. These derivatives were analogs of compounds **NE01**–**NE03**. As can be seen, the change of the substituent in the C2 position in the 1H-imidazo[4,5-f][1,10]phenanthroline ligand from 4-methylphenyl to 3-pyridine caused slight changes in the values of positive potentials of the presented complexes [41]. In 2021, a ruthenium complex in the neutral (**E**) and ionic (**ECH_3_^+^**) forms and a two-core ruthenium and silver complex (**E_2_Ag^+^**) were compared [23]. The single-core complexes behaved like previously described complexes. The two-core complex, however, proved surprising. The voltammogram did not differ from the other complexes in the first scan. However, a sharp peak at +0.49 V was observed in subsequent scans. The authors attributed this peak to the oxidation of metallic Ag(0) nanoclusters on the working electrode after reduction of **E_2_Ag^+^** in solution. Adding the ionic methyl moiety to complex **E** and then generating **E_2_Ag^+^** via Ag^+^ complexation resulted in slight stabilization of the HOMO and LUMO [23]. For compounds **D1**–**D3**, an irreversible oxidation peak at 1.87 V associated with the 1H-imidazo[4,5-f][1,10]phenanthroline ligand was detected. Several reversible and quasi-reversible reduction peaks were observed in the cathodic region at −1.21 V to −1.6 V, which can be attributed to the reduction processes occurring on the polypyridyl ligands [44]. In 2024, a dinuclear ruthenium complex linked by only one imp ligand (**D**) was studied for the first time. The way the metal centers are connected is noteworthy. The first is attached to the imp at the N^N position, while the second is attached at the N1 position in the imidazole ring and via a nitrogen atom in the pyridine substituent. The studies indicate that the oxidation process occurs at the metal centers of the Ru(III) species. Interestingly, however, a further oxidation peak was observed for compound **D**. The subsequent oxidation, occurring at a higher potential, is irreversibly attributed to the 1H-imidazo[4,5-f][1,10]phenanthroline ligand. Due to electronic communication between the two metal centers via the π-conjugated, bis-bidentate imp ligand, performance issues can be expected in the D-based LEC device. All complexes described in this article exhibited reduction processes corresponding to the ligands. B. N. Bideh et al. found that the lowest negative reduction potential depends on the imp ligand, which reflects the stabilization of the π* orbital of this ligand, probably due to its extensive π-conjugation system [37]. The authors concluded that reversible redox processes indicate good electrochemical stability of the presented complexes, meaning that both holes and electrons can be efficiently transported. This fact is beneficial for obtaining efficient and stable iTMC-LECs. A significant anodic shift is observed when comparing **M1**, **M2**, and **D** to [Ru(bpy)_3_]^2+^. This may be due to the presence of electron-donating groups on the periphery of the ligands. Furthermore, the energy gap values for **M1**, **M2**, and **D** were significantly smaller than for [Ru(bpy)_3_]^2+^ due to the stabilization of the LUMO levels and the destabilization of the HOMO levels by the ligands in the **M1**, **M2**, and **D** complexes.

## 4. Optical Properties

Before examining any compound in the emissive layer of a constructed LEC device, its physicochemical properties should be thoroughly understood. Luminescence studies in both solution and a solid state help us assess the suitability of the obtained molecules. Detailed characterization of the compounds, including determining parameters such as band maxima, lifetimes, and quantum yields (QYs), allows us to determine the behavior of the molecules in terms of optical properties. Iridium and ruthenium complexes containing 1H-imidazo[4,5-f][1,10]phenanthroline derivatives described in the literature were measured in samples at a concentration of 10^−5^ M [30]. Solvents such as dichloromethane [43] and acetonitrile [30] were used in the measurements. The emission quantum yields (Φp) were calculated by comparison with quinine sulfate (Φp = 0.545) in 1M H_2_SO_4_ (estimated error of ±5%) [30]or 0.5M H_2_SO_4_ [43]. All collected data are presented in Table 2.

In 2012, research on Ir complexes containing imp as an auxiliary ligand began [43]. Such a complex (named **1**) was observed to have two absorption bands. The lowest-energy electronic band above 450 nm was assigned to the (Ir–phenyl)-to-imp charge transfer transition. The bands with intense absorption above 450 nm were designated as MLCT/ILCT and ligand π–π* transitions [43]. This complex also showed yellow-orange phosphorescence in argon-saturated dichloromethane solution. The emission maximum was detected at 583 nm. **1** was characterized by a good quantum yield (QY) of 43% and a lifetime of 910 ns. In case **1**, a single emissive center was observed in the solution [43]. This is due to the single exponential function obtained in the lifetime measurement. Furthermore, it can be assumed that 1 emits from a charge-transfer [(Ir–phenyl)-to-imp] excited state. This fact is confirmed by the lack of vibronic structure in the phosphorescence spectrum and the relatively short calculated radiation lifetime [43]. The next Ir complexes studied under the LEC umbrella were **Ir1**, **Ir2**, and **Ir3^+^** [30]. They exhibited absorption bands in three ranges. The first one (between 260 and 300 nm) includes ligand-centered (LC) spin-allowed ^1^π-π* transitions. These bands are associated with the imp ligand and ppy ligands. Absorption bands between 300 and 430 nm are spin-allowed metal-to-ligand (^1^MLCT) and ligand-to-ligand charge transfer (^1^LLCT) transitions. The third band, with the lowest intensity, is attributed to spin-forbidden ^3^MLCT, ^3^LLCT, and LC ^3^π-π* transitions of the complexes. The authors observed that **Ir2** is bathochromically shifted relative to **Ir1** in the lowest-energy absorption band [30] (Table 2). This is a direct result of the change in the HOMO/LUMO energy gap caused by the presence of electron-withdrawing (Br) and electron-donating (OCH_3_) groups on the PI ligand of the complexes. The emission maxima of all complexes are 580, 592, and 602 nm and are attributed to the transition from the ^3^CT states. The introduction of electron-withdrawing and electron donor groups on the aryl ring of the PI ligand exerts a negligible influence on the maximum luminescence. It was also observed that **Ir2** is bathochromically shifted relative to the [Ir(ppy)_2_(phen)]PF_6_ standard. B. Bideh et al. found that this is due to the electron-deficient nature of the fused imidazole moiety with Br as an electron-withdrawing group (L2), and π-expanded structure of the PI ligand [30]. This effect stabilizes the LUMO orbital and reduces the optical gap in this compound. The opposite effect is also observed in **Ir1**. Due to the two donor groups (OCH_3_) in the L1 ligand, the LUMO orbital is destabilized, resulting in a lower maximum emission in this series. Moreover, the **Ir1**, **Ir2**, and **Ir3^+^** complexes exhibited higher quantum yields compared to the [Ir(ppy)_2_(phen)]^+^ reference. This is due to the rigid structure of the imp ligand. Thin-film studies were also performed to verify the potential application of these complexes in LEC devices. It was demonstrated that introducing the imp ligand into the complex structure results in lower intermolecular interactions in **Ir1** and **Ir2** compared to the parent complex. Comparing the QY values obtained in thin films, it was observed that those of **Ir1** and **Ir2** are approximately 2.5 to 3 times higher than [Ir(ppy)_2_(phen)]PF_6_. This proves that the bulky phenyl groups at the PI ligands significantly suppress the phosphorescence concentration quenching [30]. In 2024, research was conducted on dinuclear Ir(III) complexes. The studies revealed that their absorption and emission properties (transition characteristics) are very similar to those of complexes already known from the literature (Table 2). Furthermore, substitution of the imidazole moiety at the C2 position does not significantly affect the electronic properties of this type of emitter [38].

In 2016, ruthenium(II) complexes (having 1H-imidazo[4,5-f][1,10]phenanthroline derivatives as ligands) were described in the literature for the first time and tested as emitters in LECs [45]. 2-(2-hydroxyphenyl)-1-(4-bromophenyl)1H-imidazo[4,5-f][1,10]phenanthroline, abbreviated as hpbpip, was selected as the first ligand from the group of 1H-imidazo[4,5-f][1,10]phenanthroline derivatives. The compounds tested with the considered ligand showed absorption bands at 460 nm, 330 nm, and 275 nm. The lowest-energy band is assigned to a spin-allowed metal-to-ligand charge-transfer (MLCT) transition due to an overlapping of dπ(Ru) → π*(bpy or phen) and dπ(Ru) → π* (hpbpip) transitions [45]. The next band (330 nm) was assigned to intraligand bands from the 1H-imidazo[4,5-f][1,10]phenanthroline ligand. The last high-intensity band comes from the polypyridyl π → π* transition. In comparing the **NE1**–**NE4** complexes to [Ru(bpy)_3_]^2+^, a bathochromic shift can be observed. This is due to the larger range of π delocalization in the 1H-imidazo[4,5-f][1,10]phenanthroline ligand. The emission maximum for **NE1**–**NE4** was in the range of 610–635 nm. The most red-shifted compound was **NE2** (Table 3). Interestingly, **NE1** exhibited a significantly higher photoluminescence quantum yield (QY) than the standard. The authors concluded that this beneficial effect results from the attachment of hpbpip to the [Ru(bpy)_3_]^2+^ structure [45]. In the same year, compounds **NE01**–**NE04** containing 2-(4-methylphenyl)-1-(4-methoxyphenyl)-1H-imidazo[4,5-f][1,10]phenanthroline (MPIP) in their structure were also studied [19]. Compared to **NE1**–**NE4**, they exhibited only two absorption band ranges. These were the bands between 220 nm and 290 nm associated with the π-π* transition of the ligands and a broad band around 460 nm corresponding to the ^1^MLCT transition. The authors assumed that the broadening of the ^1^MLCT band results from the overlap of the dπ(Ru) → π*(bpy or phen) and dπ(Ru) → π*(MPIP) transitions. Furthermore, introducing the imp ligand into the complex structure caused a red shift in the absorption maxima relative to [Ru(bpy)_3_]^2+^. This is due to the π-extended structure of the 1H-imidazo[4,5-f][1,10]phenanthroline ligand. The **NE01**–**NE04** complexes showed similar emission in solution around 600 nm (Table 3). Comparison of the photoluminescence spectra in solution and a solid thin film indicates that the emission maximum strongly depends on the molecular environment, which can probably be attributed to the polarity effects of the medium [19]. In 2017, B. Bideh et al. described binuclear complexes (**B1** and **B2**) [42]. These complexes are linked by a common substituent at the N1 position of the imp ligand. Interestingly, these compounds exhibited the same absorption and emission characteristics as the single-nuclear complexes [42]. In 2018, compounds **B1** and **B2** were compared with the newly synthesized **B01**–**B03**. The main difference between them was the method of linkage using the imp ligand. The authors showed that **B01**–**B03**, linked together by a phenyl substituent at the C2 position, did not change the shape of the absorption spectra compared to **B1**–**B2**. In comparison with the binuclear complexes containing non-conjugated complexes, **B01** and **B02** showed more intense absorption bands, which can be attributed to the increasing of π-conjugation of the system [40]. **B01**–**B03** were characterized by an intense emission band around 630 nm. They exhibited a bathochromic shift compared to compounds **B1** and **B2**. In 2020, research was conducted on **Bn1**–**Bn3** [41]. As for compounds previously described in the literature, two regions were observed in the UV-VIS spectra. The first, with a band around 300 nm, can be attributed to intraligand charge transfer (ILCT). The second, in the visible region (around 450 nm), is related to metal-to-ligand charge transfer (MLCT) [41]. The emission band maxima in solution for **Bn1**–**Bn3** were in the range of 608–634 nm. Compared to **NE01**–**NE03**, known from the literature, a clear red shift in the band is visible. Replacing the 4-methylphenyl substituent with a 3-pyridine substituent in the imp ligand caused a favorable bathochromic shift and allowed for higher luminescence quantum yields for the entire series of compounds to be obtained [41]. This effect was also observed in solid states. A novelty in the research was the measurement of ruthenium complexes in the ionic forms (**E**) and (**ECH_3_^+^**), as well as an ionic dinuclear ruthenium–silver complex (**E_2_Ag^+^**). Interestingly, the ionic ligand substituent was found to have no significant effect on the optical properties [23]. B. Bideh et al. observed that the introduction of an ionic moiety into the complex reduces aggregation and increases the quantum emission efficiency, which is promising for semiconductor light-emitting devices [23]. In the case of **D1**–**D3**, during photoluminescence studies, it was observed that the coupling of two ruthenium(II) centers with a bridging imp ligand increased the total number of non-radiative paths and consequently shortened the luminescence lifetime in the excited state compared to [Ru(bpy)_3_]^2+^ [44]. In 2024, B. Bideh et al. studied complexes **D**, **M1**, and **M2** [37]. In the case of **M1** and **M2**, the obtained results were typical for ruthenium complexes with 1H-imidazo[4,5-f][1,10]phenanthroline derivatives. However, the situation was more interesting for **D**. This complex was distinguished by how ruthenium was attached to the imp ligand. An additional shoulder around 330 nm was observed in the UV-Vis spectra, corresponding to the intraligand transition from the bidentate imp ligand. Interestingly, **D** showed a significant decrease in photoluminescence quantum yield compared to **M1** and **M2** (Table 3). This behavior differs from the binuclear complexes based on the 1H-imidazo[4,5-f][1,10]phenanthroline ligand known from the literature. The authors attributed this to the severe self-quenching of emission due to the decrease in the internuclear distance [37].

**Figure 2:**
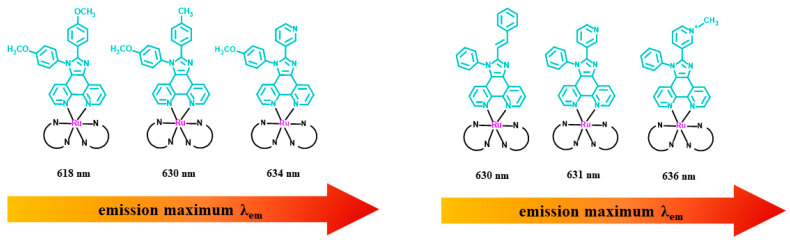
The influence of the substituent in the 1H-imidazo[4,5-f][1,10]phenanthroline derivative on the bathochromic emission shift.

Considering ruthenium(II) complexes, it can be observed that changing the substituent at the C2 position in the 1H-imidazo[4,5-f][1,10]phenanthroline ligand influences the bathochromic shift in the emission spectra of the complexes (Figure 2). This shows that these ligands significantly improve the luminescent properties of the presented compounds. Moreover, all complexes containing the imp ligand in their structure exhibited better optical parameters than the **[Ru(bpy)_3_]^2+^** standard.

## 5. Device Properties

There is a constant search for new technological solutions for light sources. LECs are one of the most promising devices, and numerous studies are being conducted on this aspect. The decisive advantage of electrochemical cells emitting light is their straightforward construction [19,23,30,36,37,38,39,40,41,42,43,44,45]. Therefore, the results for LECs with the discussed iridium(III) and ruthenium(II) complexes with 1H-imidazo[4,5-f][1,10]phenanthroline ligands in the emission layer are presented further on in the chapter [19,23,30,36,37,38,39,40,41,42,43,44,45]. In the case of the LEC design under consideration, the first element was the anode, which was glass coated with indium tin oxide (ITO). The anode was then cleaned in an ultrasonic bath and specially dried [19,23,30,36,37,38,39,40,41,42,43,44,45]. Then, a layer of PEDOT:PSS (poly(3,4-ethylenedioxythiophene):poly(styrenesulfonate)) was applied to the prepared ITO plate using the spin method [30,36,37,38,43,44]. The next (emissive) layer consisted of iridium(III) or ruthenium(II) complexes. In the emissive layer, devices containing iridium(III) complexes also had an ionic liquid added. The most commonly used ionic liquid was 1-butyl-3-methylimidazolium hexafluorophosphate ([BMIM^+^] [(PF_6_)^−^]) [30,38,43]. In selected cases, 1-ethyl-3-methylimidazolium hexafluorophosphate ([EMIM^+^] [(PF_6_)^−^]) was also used [36]. The only deviation from the above-described procedure was in that used for ruthenium(II) complexes, as the obtained compounds were directly spin-coated as thin layers onto the ITO surface [19,23,39,40,41,42,45]. The final layer of the LEC was the cathode. Al [30,43], Ag [37,38,44], or Ga:In eutectic [19,23,36,39,40,41,42,45] was used as the cathode. Finally, the ruthenium(II) complexes were sealed with epoxy cement [19,23,39,40,41,42,45]. A vital aspect distinguishing LECs from OLEDs is that these devices do not require encapsulation and can be characterized at room temperature. All results for LECs are presented in Table 4.

The first Ir(III) complex containing a 1H-imidazo[4,5-f][1,10]phenanthroline derivative in its structure, studied in the context of an LEC, was compound 1. In addition to derivative **1**, the prototype device also contained an ionic liquid in the emission layer. This addition was intended to shorten the device’s turn-on time [43]. The obtained electroluminescence spectrum corresponds to the photoluminescence of this compound in a thin film. Compound **1** emits orange, as indicated by its CIE coordinates (Table 4). The authors noted that the device’s response is rapid. The presented prototype achieved a luminance of 100 cd/m^2^ in just 45 s. The highest luminance of 684 cd/m^2^ was obtained in 28 min. The device’s maximum efficiency was 6.5 cd/A, 1.8 times higher than the analogs described in the literature [43]. Furthermore, stability testing of the device showed that its luminance and efficiency decreased by only 20% after 850 h (35 days) of continuous operation. Another attempt to use Ir(III) complexes with an imp derivative for LEC research was reported in 2023. The maximum emission and electroluminescence (EL) spectra were very similar to those of these complexes in thin films. Based on this, it can be concluded that the nature of the EL and PL emission is identical and originates from the same excited state in both excitation methods [30]. **Ir1**, **Ir2**, and **Ir3^+^** exhibited emission from yellow to orange with coordinates (0.52, 0.48), (0.64, 0.35), and (0.61, 0.39). The shortest wavelength was observed for compound **Ir1** (Table 4). This is due to the influence of electron-donating and electron-withdrawing groups on the 1H-imidazo[4,5-f][1,10]phenanthroline ligand, effectively changing the LUMO levels in this series. Under constant current of 100 A/m^2^, the LECs based on **Ir1**, **Ir2**, and **Ir3^+^** offer maximum luminance of 870, 563, and 45 cd/m^2^ and external quantum efficiency (EQE) of 3.1, 2.5, and 0.24%, respectively [30]. In comparing the presented complexes with the [Ir(ppy)_2_(N^N)]^+^ (named **S1**–**S4**) emitters described in the literature with a N^N phenanthroline-based auxiliary ligand, it can be observed that **Ir1** presents the highest L_max_ and EQE values. The prototype LEC devices based on **Ir1**, **Ir2**, and **Ir3^+^** showed a fast response, with response times of 0.65, 1.3, and 0.15 h, respectively. The significant improvement in the response time of the **Ir3^+^**-based LEC device can therefore be attributed to the ionic nature of the emission layer resulting from the accelerated formation of doped regions [30]. This demonstrates the crucial role of the ionic methylpyridinium fragment in reducing the tone of LEC devices. Moreover, the potential of modifying the imp ligand with ionic groups can be observed. The authors demonstrated that, compared to the parent archetype [Ir(ppy)_2_(phen)]^+^, replacing phenanthroline with the ligand 1H-imidazo[4,5-f][1,10]phenanthroline leads to an impressive improvement in the EL properties of LEC devices with the same structure [30]. In 2024, M. Mazaheri presented dinuclear iridium(III) compounds [38]. The authors compared the obtained results with the model complex [Ir(ppy)_2_(phen)]PF_6_. Devices fabricated based on **IrL1**–**IrL2**, measured under the same conditions as the standard, showed an 8–10-fold increase in maximum brightness. The significant improvement in L_max_ is attributed to the increased luminescence efficiency due to the rigid structure of the imp ligand. The authors demonstrated that cationic dinuclear iridium(III) complexes based on sterically hindered bulky bridging 1H-imidazo[4,5-f][1,10]phenanthroline ligands help in obtaining highly efficient semiconductor lighting devices, such as LECs and OLEDs [38].

Ruthenium(II) complexes containing 1H-imidazo[4,5-f][1,10]phenanthroline derivatives in their structure were first investigated for use as emitters in LECs in 2016 [45]. The authors of these studies reported in the article that they had obtained and fully characterized a series of ruthenium(II) complexes (**NE1**–**NE4**) differing in the structure of the auxiliary ligands. The 1H-imidazo[4,5-f][1,10]phenanthroline derivative was the same in each case [45]. Interestingly, all LECs (with **NE1**–**NE4**) exhibited luminances in the 790–2250 cd/m^2^ range. The best results were achieved by the device with the **NE2** derivative (2250 cd/m^2^). The cell containing **NE2** exhibited the highest electroluminescence quantum efficiency (EQE = 0.61%) and the lowest turn-on voltage (V_on_ = 2.6 V). It was also characterized by a deep-red color with CIE(x, y) coordinates of (0.654, 0.344). Another group of ruthenium(II) complexes containing 1H-imidazo[4,5-f][1,10]phenanthroline derivatives (**NE01**–**NE04**) was also tested for use in LECs in 2016 [19]. For these compounds, the authors demonstrate that the presence of methyl groups in the ligands causes a lower turn-on voltage. This effect is particularly observed for the **NE02** complex, for which the V_on_ decreased to 2.3 V. For comparison, the turn-on voltage for **NE04**, which contains two bathophenanthroline molecules, is 3.1 V. Furthermore, the authors indicate that the low turn-on voltage for ruthenium(II) complexes is also due to the presence of the counterion (ClO_4_)^−^, which can easily move through the emission layer [19]. This phenomenon may be because counterions (ClO_4_)^−^ migrate to the positive electrode (anode) when an electric field is applied. They then accumulate at the phase boundary, leaving a positive charge density on the sides of the negative electrode [19]. As the number of accumulated ions increases, the energy barrier at the interface decreases and thus the number of injected electron charges increases [19]. Moreover, the authors also prove that the **NE04** device is characterized by high efficiency (0.45 cd/A) and high electroluminescence quantum efficiency (EQE = 1.367%), compared to other LECs containing ruthenium(II) complexes in the emission layer. Among the tested cells, the **NE02** device also deserves a special mention, as it exhibits a luminance of 2395 cd/m^2^ [19]. Another literature report on ruthenium(II) complexes with ligands based on 1H-imidazo[4,5-f][1,10]phenanthroline was published in 2017 [42]. In this work, two dinuclear ruthenium(II) complexes (**B1**–**B2**) were investigated for the first time for use as emitters in LECs. The double-derivative 1H-imidazo[4,5-f][1,10]phenanthroline, linked at the N1 position of the central imidazole ring, was the ligand, enabling the formation of the dinuclear complexes. Comparing the described dinuclear ruthenium(II) complexes, we can successfully observe that compound **B2** is significantly better than **B1** in terms of parameters such as the following: turn-on voltage (**B1** = 4.5 V, **B2** = 3.1 V), luminance (**B1** = 193 cd/m^2^, **B2** = 742 cd/m^2^), lifetime (**B1** = 539s, **B2** = 1104s), and electroluminescence quantum yield (**B1** = 0.14%, **B2** = 0.68%) [42]. The difference in parameters is due to the use of an auxiliary ligand in the form of 4,4′-dimethyl-2,2′-bipyridine (dmbpy) [42]. The research on dinuclear ruthenium(II) complexes was continued in the work by B. N. Bideh et al., published in 2018 [40]. As in the case of compounds **B1**–**B2**, the dinuclear characteristic of complexes **B01**–**B03** was achieved with the 1H-imidazo[4,5-f][1,10]phenanthroline derivative. However, this time, the ligand was linked at the C2 position of the central imidazole ring via a phenyl ring. This preserved the aromatic characteristics of the ligand. Considering the above, the authors argued that the unconjugated bridging ligand (in complexes **B1**–**B2**) increases the degree of rotational freedom of the molecules, causing disorder in the solid layer [40]. This translates into reduced molecular disorder in the solid state of the device. Furthermore, separating adjacent metal centers in the dinuclear ruthenium(II) complexes containing a wholly aromatic bridging ligand likely prevents molecular aggregation in the emissive layer [40]. The authors’ conclusion indicates that the aromaticity of the 1H-imidazo[4,5-f][1,10]phenanthroline derivative improves the photophysical properties of the LEC. This is due to the increased homogeneity of the emission layer in the device [40]. Comparing the compounds under consideration (**B01**–**B03**) with each other, we can see that the **B02** derivative exhibits the best parameters in the LEC. This applies to turn-on voltage, luminance, and electroluminescence quantum efficiency. The device containing **B02** in the emission layer is also better than the **B2** cell in terms of parameters such as EQE, V_on_, and t_1/2_ [40]. The next article on single-core ruthenium(II) complexes with ligands based on 1H-imidazo[4,5-f][1,10]phenanthroline derivatives was published in 2019 [39]. The compounds (**F1**–**F3**) presented in the publication were structurally diverse in terms of the auxiliary ligand (**F1**—2,2′-bipyridine, **F2**—4,4′-dimethyl-2,2′-bipyridine, **F3**—1,10-phenanthroline). The 1H-imidazo[4,5-f][1,10]phenanthroline derivative was the same in the three cases. The authors demonstrated that devices constructed based on the considered ruthenium(II) complexes are characterized by low turn-on voltages, specifically 2.4 V for **F1**, 2.3 V for **F2**, and 2.8 V for **F3**. According to the researchers, such low V_on_ values result from the enriched electron density of the complexes [39]. This means that the single-core architecture of ruthenium(II) complexes is also extremely attractive in designing emitters for LECs. Furthermore, in 2020, B. Bideh and H. Shahroosvand investigated how changing the substituent at the C2 position of the 1H-imidazo[4,5-f][1,10]phenanthroline derivative would affect the properties of ruthenium(II) complexes and, consequently, LEC devices [41]. The **Bn1**–**Bn3** series included ruthenium(II) complexes with 1-(4-methoxyphenyl)-2-(pyridin-3-yl)-1H-imidazo[4,5-f][1,10]phenanthroline. The obtained results were compared with compounds **NE01**–**NE04**, previously known from the literature. The authors observed that changing the substituent from methylphenyl to pyridine causes a significant red shift in the EL emission. Comparing the obtained parameters, such as electroluminescence quantum efficiency (EQE), one can observe an improvement in the device properties resulting from the change of the substituent at the C2 position. The authors speculated that the reason for this situation may be the fact that the nitrogen atom of protonated pyridine can react with the counterion (ClO_4_)^−^ [41]. As a result, a Lewis acid–base adduct (pyH^+^:ClO_4_^−^) can form in the inner coordination sphere of the complex. Consequently, both the inner and outer coordination spheres contribute to charge accumulation. In the **NE01**–**NE03** complexes, only the outer coordination sphere contributes to charge accumulation. This may indicate that in the **Bn1**–**Bn3** series, three counterions (ClO_4_)^−^ contribute to charge transfer between electrodes. In comparison, in the **NE01**–**NE03** complexes, only two (ClO_4_)^−^ do [41]. In 2021, research was conducted on compounds **E**, **ECH_3_^+^**, and **E_2_Ag^+^**. The novelty of this research was the creation of an LEC without using any ionic additive or conductive polymer [23]. All tested compounds exhibited EL emission maxima in the deep-red (DR) region (Table 4). Compared to the DR emitters reported so far in the literature, all three complexes exhibited a low turn-on voltage of approximately 2.3 V. Of the three, **ECH_3_^+^** exhibited the worst V_on_ (Table 4), which can be attributed to the electron-withdrawing nature of the ionic moiety of PyCH_3_^+^ located on the imp ligand. The authors demonstrated that the device with **E_2_Ag^+^** in the emissive layer achieved the best performance in terms of turn-on time (t) and electroluminescence quantum efficiency (EQE). In comparing ionic complexes with **E**, it can be observed that the high mobility of counterions in the emissive layer resulted in higher current densities and shorter response times. Surprisingly, the turn-on time, which is defined as the time to reach maximum luminance at a voltage of 5 V, was drastically reduced from 86s for **E** to 27 s and 12 s for **ECH_3_^+^** and **E_2_Ag^+^**, respectively [23]. The promising results of the **E**, **ECH_3_^+^**, and **E_2_Ag^+^** series led to further studies with the 1H-imidazo[4,5-f][1,10]phenanthroline ligand with a pyridine or methylpyridine substituent at the C2 position. This time, B. Bideh et al. decided to examine the behavior of dinuclear complexes. The **D1**–**D3** series showed EL emission in the near-infrared region with coordinates around (0.73, 0.26). Interestingly, the similar EL maximum in the mono- and dinuclear complexes indicates that the methylene groups (–CH_2_–) in the bridging 1H-imidazo[4,5-f][1,10]phenanthroline ligand tend to isolate the electronic coupling between the two ruthenium centers in the solid phase. Therefore, there is no electronic interaction between them [44]. All complexes exhibited very low turn-on voltages (Table 4). The **D1**–**D3** series exhibited significantly longer lifetimes (t_1/2_) than similar single-core complexes [45]. This is due to the imp ligand, which prevents degradation of the emitter in an electric field and hinders the penetration of nucleophilic molecules, such as water. The authors also observed that uncoordinated pyridine groups in the 1H-imidazo[4,5-f][1,10]phenanthroline derivative ligand increase the emission stability of the cells [44]. In 2024, the authors focused on the role of emitter molecule substitution in LEC efficiency. Compound **D** was particularly interesting in their study due to its coordination with the metal center. It turned out that it exhibited low EL emission intensity and low LEC device efficiency. Case **D** shows that the dual-core strategy is not always effective in improving cell efficiency. Furthermore, molecular engineering of the dual-core complex is also crucial [37].

## 6. Conclusions

Light-emitting electrochemical cells (LECs) are an exciting alternative to many currently used and researched lighting technologies. Their straightforward design and the ability to use air-stable electrodes make this solution competitive in terms of the product’s initial price and operational life. The weakest element of LECs is currently the emitters used in the active layer. The current number of emitters studied and described in the literature that achieve satisfactory parameters is limited. Furthermore, knowledge about the design of such compounds is also restricted. However, in recent years, reports have appeared in the literature on the use of iridium(III) and ruthenium(II) complexes with 1H-imidazo[4,5-f][1,10]phenanthroline-derived ligands in the LEC emission layer. In many cases, the ligands considered have been extremely promising in both synthesis and the final physicochemical properties of the emitters studied. Therefore, this literature review summarizes the current knowledge regarding the presented iridium(III) and ruthenium(II) complexes. We hope that it will help in designing and testing further emitters for LECs. Also, we can safely assume that further expansion of the substituents at the N1 and C2 positions in the case of 1H-imidazo[4,5-f][1,10]phenanthroline derivatives may prove highly beneficial in terms of the physicochemical properties and potential applications of the new single- and double-core iridium(III) and ruthenium(II) complexes in LECs. In particular, if these were extensive aromatic or heteroaromatic substituents, they would significantly impact the physicochemical properties of the ligand and the final complex. Further modification of ligands containing two 1H-imidazo[4,5-f][1,10]phenanthroline cores may prove highly beneficial. Enriching compounds of this type with additional aromatic fragments or functional groups can significantly affect the parameters of the iridium(III) and ruthenium(II) complexes under consideration. Furthermore, structural modification of auxiliary ligands such as 2,2′-bipyridine, 4,4′-dimethyl-2,2′-bipyridine, 1,10-phenanthroline, and 4,7-diphenyl-1,10-phenanthroline is an exciting direction of research.

## Figures and Tables

**Table 1:** Electrochemical data.

Code	Solvent	E_1/2ox_ [V] (ΔE [mV])	E_1/2red_ [V] (ΔE [mV])	HOMO [eV]	LUMO ^a^ [eV]	Eg [eV]	Ref.
Iridium(III) complexes							
**1**	MeCN	0.88 (83)	−1.76 (73)	−	−	2.64 *	[43]
	DMF	0.95	−1.73 (78)	−	−	2.68 *	[43]
**Ir1**	MeCN	0.87 (81)	−1.81 (70)	−5.67	−2.99	2.68	[30]
**Ir2**	MeCN	0.91 (86)	−1.61 (74)	−5.71	−3.19	2.52	[30]
**Ir3^+^**	MeCN	0.89 (112)	−1.70 (106)	−5.69	−3.10	2.59	[30]
**IrL1**	MeCN	1.34 (193)	−1.31 (200)	−6.14	−3.49	2.65	[38]
**IrL2**	MeCN	1.35 (95)	−1.27 (105)	−6.15	−3.53	2.62	[38]
**C1**	MeCN	0.88 ^a^	−1.81	−	−	2.69	[36]
**C2**	MeCN	0.89 ^a^	−1.81	−	−	2.70	[36]
**C3**	MeCN	0.88 ^a^	−1.80	−	−	2.68	[36]
**C4**	MeCN	1.10 ^a^	−1.74	−	−	2.84	[36]
**C5**	MeCN	1.11 ^a^	−1.75	−	−	2.86	[36]
**C6**	MeCN	1.12 ^a^	−1.74	−	−	2.86	[36]
Ruthenium(II) complexes							
**NE1**	MeCN	1.38 (70)	−1.02	−5.82	−3.38	2.44	[45]
**NE2**	MeCN	1.32 (60)	−0.88	−5.77	−3.52	2.25	[45]
**NE3**	MeCN	1.36 (105)	−0.86	−5.81	−3.54	2.27	[45]
**NE4**	MeCN	1.33 (126)	−0.96	−5.78	−3.44	2.34	[45]
**NE01**	MeCN	1.31 (75)	−1.33	−5.68	−3.04	2.64	[19]
**NE02**	MeCN	1.24 (84)	−1.23	−5.61	−3.14	2.47	[19]
**NE03**	MeCN	1.31 (63)	−1.40	−5.68	−2.97	2.71	[19]
**NE04**	MeCN	1.28 (71)	−1.25	−5.65	−3.12	2.53	[19]
**B1**	MeCN	1.30 (60)	−	−5.67	−3.34 ^b^	2.33	[42]
**B2**	MeCN	1.27 (84)	−	−5.64	−3.37 ^b^	2.27	[42]
**B01**	MeCN	1.28 (60)	−	−5.65	−3.41 ^b^	2.24	[40]
**B02**	MeCN	1.21 (90)	−	−5.58	−3.36 ^b^	2.22	[40]
**B03**	MeCN	1.31 (68)	−	−5.70	−3.37 ^b^	2.33	[40]
**F1**	MeCN	1.30 (70)	−	−5.67	−3.37 ^b^	2.30	[39]
**F2**	MeCN	1.20 (81)	−	−5.57	−3.33 ^b^	2.24	[39]
**F3**	MeCN	1.31 (80)	−	−5.68	−3.32 ^b^	2.36	[39]
**Bn1**	MeCN	1.29 (98)	−	−5.66	−3.41 ^b^	2.25	[41]
**Bn2**	MeCN	1.21 (91)	−	−5.58	−3.36 ^b^	2.22	[41]
**Bn3**	MeCN	1.33 (100)	−	−5.70	−3.43 ^b^	2.27	[41]
**E**	MeCN	1.21 (71)	−	−5.58	−3.37 ^b^	2.21	[23]
**ECH_3_^+^**	MeCN	1.26 (93)	−	−5.63	−3.39 ^b^	2.24	[23]
**E_2_Ag^+^**	MeCN	1.31 (90)	−	−5.68	−3.46 ^b^	2.22	[23]
**D1**	MeCN	1.33 (70)	−1.22 (90)	−5.75	−3.20	2.55	[44]
**D2**	MeCN	1.25 (87)	−1.23 (130)	−5.67	−3.19	2.48	[44]
**D3**	MeCN	1.28 (85)	−1.23 (80)	−5.71	−3.20	2.51	[44]
**M1**	MeCN	1.21 (89)	−1.18	−5.58	−3.36	2.22	[37]
**M2**	MeCN	1.25 (87)	−1.23	−5.60	−3.31	2.29	[37]
**D**	MeCN	1.22 (95)	−1.22	−5.59	−3.34	2.25	[37]
[Ru(bpy)_3_]^2+^	MeCN	1.29 (0.079)	−1.31	−5.68	−3.08	2.60	[37]

^a^ Measurements made using Fc^+^/Fc as the standard; * Eg = E_1/2ox_ − E_1/2red_, ^b^ E_LUMO_ = E_HOMO_ + E_0–0_, E_0–0_ was calculated from the intersection of absorption and emission spectra.

**Table 2:** Luminescent properties of iridium(III) complexes.

Code	Solvent	Absorbance	Emission					Eg_opt_ [eV]	Ref.
		λ_abs_ [nm] (log ε · 10^3^)	Solution				Film		
			λ_em_ [nm] (φ)	τ [ns]	k_r_ [s^−1^]	k_nr_ [s^−1^]	λ_em_ [nm] (*φ*)		
**1**	CH_2_Cl_2_	274 (84), 299 (54), 388 (10), 405 (9.5), 467 (1.4)	583 (0.43)	910	4.7 × 10^5^	6.3 × 10^5^	590 (0.45)	-	[43]
**Ir1**	MeCN	269 (84.2), 294 (58.3), 334 (22.1), 376 (10.8), 396 (10.0), 466 (1.4)	580 (0.46)	880	5.20 × 10^5^	6.14 × 10^5^	584 (0.32)	2.54	[30]
**Ir2**	MeCN	274 (82.0), 302 (50.5), 331 (35.7), 383 (12.4), 402 (11.4), 482 (1.6)	602 (0.42)	970	4.33 × 10^5^	5.98 × 10^5^	606 (0.26)	2.45	[30]
**Ir3^+^**	MeCN	272 (78.0), 303 (4.89), 318 (32.8), 341 (16.0), 383 (7.8), 395 (7.5), 452 (0.6)	592 (0.40)	950	3.66 × 10^5^	6.42 × 10^5^	603 (0.08)	2.52	[30]
**IrL1**	CH_2_Cl_2_	274 (5.70), 297 (5.60), 344 (5.00), 345 (5.00), 387 (4.80), 475 (3.97)	570 (0.27)	790	3.4 × 10^5^	9.2 × 10^5^	590 (0.31)	2.58	[38]
**IrL2**	CH_2_Cl_2_	268 (5.75), 297 (5.58), 344 (5.00), 343 (5.00), 387 (4.88), 475 (3.98)	582 (0.36)	850	4.2 × 10^5^	7.5 × 10^5^	598 (0.39)	2.55	[38]
**C1**	MeCN	276, 297, 345, 384, 403	602 (0.18)	630	−	−	−	−	[36]
	CH_2_Cl_2_	278, 298, 346 388, 412	574 (0.35)	890	−	−	−	−	[36]
**C2**	MeCN	279, 294, 381, 403	600 (0.16)	580	−	−	−	−	[36]
	CH_2_Cl_2_	280, 299, 389, 414	576 (0.36)	960	−	−	−	−	[36]
**C3**	MeCN	274, 295, 341, 381, 406	603 (0.18)	530	−	−	−	−	[36]
	CH_2_Cl_2_	276, 296, 345, 387, 412	588 (0.31)	770	−	−	−	−	[36]
**C4**	MeCN	277, 300, 347, 378	528 (0.39)	4660	−	−	−	−	[36]
	CH_2_Cl_2_	278, 303, 352, 387	522 (0.60)	3950	−	−	−	−	[36]
**C5**	MeCN	279, 301, 349, 384	528 (0.27)	5670	−	−	−	−	[36]
	CH_2_Cl_2_	281, 304, 358, 390	525 (0.61)	5020	−	−	−	−	[36]
**C6**	MeCN	277, 301, 346, 379	527 (0.35)	4990	−	−	−	−	[36]
	CH_2_Cl_2_	278, 303, 355, 390	523 (0.63)	4570	−	−	−	−	[36]
**[Ir(ppy)_2_ (phen)]^+^**	MeCN		583 (0.39)	230	17.3 × 10^5^	2.7 × 10^5^	591 (0.11)	−	[30]

**Table 3:** Luminescent properties of ruthenium(II) complexes.

Code	Solvent	Absorbance		Emission		Ref.
		λ_abs_ [nm] (log ε · 10^3^)		λ_em_ [nm] (φ)		
		Ligand Transitions	MLCT	Solution	Film	
**NE1**	MeCN	220 (4.69), 284 (4.97), 323 (4.40)	455 (4.24)	618 (0.121)	626	[45]
**NE2**	MeCN	220 (4.96), 282 (4.94), 318 (4.47)	469 (4.20)	635 (0.096)	676	[45]
**NE3**	MeCN	224 (4.89), 262 (4.97), 337 (4.19)	454 (4.20)	610 (0.086)	626	[45]
**NE4**	MeCN	224 (4.97), 276 (5.10), 329 (4.42)	463 (4.43)	620 (0.078)	635	[45]
**NE01**	MeCN	233 (4.61), 255 (4.10), 285 (4.98)	456 (4.25)	605 (0.119)	632 (0.162)	[19]
**NE02**	MeCN	221 (4.95), 259 (4.11), 285 (4.96)	462 (4.22)	618 (0.091)	636 (0.151)	[19]
**NE03**	MeCN	227 (4.87), 263 (4.98), 276 (4.18)	453 (4.21)	595 (0.089)	633 (0.123)	[19]
**NE04**	MeCN	228 (4.91), 279 (5.12), 309 (4.31)	464 (4.45)	609 (0.061)	643 (0.098)	[19]
**B1**	MeCN	252 (4.04), 285 (4.97)	457 (4.24)	604 (0.116)	645	[42]
**B2**	MeCN	220 (4.91), 287 (4.95)	463 (4.23)	620 (0.099)	690	[42]
**B01**	MeCN	242 (4.31), 286 (4.87), 345 (4.33)	458 (4.26)	614 (0.112)	630	[40]
**B02**	MeCN	245 (4.28), 284 (4.89), 346 (4.31)	467 (4.24)	631 (0.094)	692	[40]
**B03**	MeCN	259 (4.84), 288 (4.43), 380(4.47)	456 (4.21)	603 (0.081)	613	[40]
**F1**	MeCN	296 (4.97), 336 (4.36)	458 (4.28)	609 (0.116)	626	[39]
**F2**	MeCN	297 (4.93), 338 (4.33)	467 (4.26)	630 (0.099)	689	[39]
**F3**	MeCN	297 (4.89), 351 (4.21)	454 (4.23)	594 (0.088)	631	[39]
**Bn1**	MeCN	279 (4.87)	450 (4.31)	618 (0.122)	630 (0.168)	[41]
**Bn2**	MeCN	259 (4.79), 279 (4.96)	458 (4.33)	634 (0.096)	696 (0.153)	[41]
**Bn3**	MeCN	276 (4.41)	447 (4.23)	608 (0.092)	658 (0.129)	[41]
**E**	MeCN	206, (4.73), 285 (4.91)	464 (4.32)	631 (9.9)	691	[23]
**ECH_3_^+^**	MeCN	208 (4.75), 249 (4.71), 286 (4.90)	465 (4.33)	636 (9.6)	661	[23]
**E_2_Ag^+^**	MeCN	208 (4.76), 285 (4.92)	464 (4.31)	632 (9.8)	685	[23]
**D1**	MeCN	243 (4.45), 289 (4.86)	451 (4.07)	615 (0.101)	626 (0.093)	[44]
**D2**	MeCN	230 (4.68), 295 (4.91)	475 (4.20)	637 (0.098)	683 (0.085)	[44]
**D3**	MeCN	229 (4.78), 282 (4.89)	463 (4.21)	629 (0.097)	675 (0.056)	[44]
**M1**	MeCN	258 (4.64), 285 (4.99)	464 (4.27)	630 (11)	670 (0.16)	[37]
**M2**	MeCN	257 (4.53), 284 (4.95)	457 (4.21)	620 (9.3)	665 (0.15)	[37]
**D**	MeCN	252 (4.56), 283 (4.93)	448 (4.27)	623 (1.2>)	668	[37]
**[Ru(bpy)_3_]^2+^**	MeCN	245 (4.4), 290 (4.91)	448 (4.17)	617 (9.5)	645 (12)	[37]

**Table 4:** Parameters of LECs containing iridium(III) and ruthenium(II) complexes with 1H-imidazo[4,5-f][1,10]phenanthroline ligands.

Code	EL_max_ [nm]	V_on_ [V]	t_1/2_ [s]	t_on_ [s]	L_max_ [cd/m^2^]	E_max_ [cd/A]	J [A/m^2^]	EQE [%]	CIE [x,y]	Ref.
Iridium(III) complexes										
**1**	589	2.6	−	−	684	6.5	−	−	(0.550, 0.440)	[43]
**Ir1**	581	2.6	2130 h	0.65 h	870	8.60	−	3.1	(0.519, 0.480)	[30]
**Ir2**	605	2.7	1450 h	1.30 h	563	5.52	−	2.5	(0.648, 0.351)	[30]
**Ir3^+^**	596	2.6	2.25 h	0.15 h	45	0.38	−	0.24	(0.608, 0.391)	[30]
**IrL1**	586	3.4	−	−	208	3.46	60	−	(0.551, 0.448)	[38]
**IrL2**	598	3.6	−	−	252	4.50	56	−	(0.617, 0.381)	[38]
**C1**	596	3.5	−	−	15.5 lx	−	−	−	(0.410, 0.570)	[36]
**C2**	587	2.9	−	−	21.7 lx	−	−	−	(0.410, 0.570)	[36]
**C3**	579	3.9	−	−	16.1 lx	−	−	−	(0.410, 0.570)	[36]
**C4**	527	4.4	−	−	3.5 lx	−	−	−	(0.520, 0.470)	[36]
**C5**	523	3	−	−	4.4 lx	−	−	−	(0.520, 0.470)	[36]
**C6**	528	3.5	−	−	4.1 lx	−	−	−	(0.520, 0.470)	[36]
**[Ir(ppy)_2_(phen)]^+^**	578	−	73 h	6.4 h	63	5.8	−	2.1	−	[30]
**S1**	577	−	−	−	>1200	2.31	−	−	−	[30]
**S2**	600	−	65 h	−	About 27	−	−	−	−	[30]
**S3**	578	−	230 h	13 h	92	5.2	−	−	−	[30]
**S4**	589	−	2000 h	0.47 h	689	6.5	−	−	−	[30]
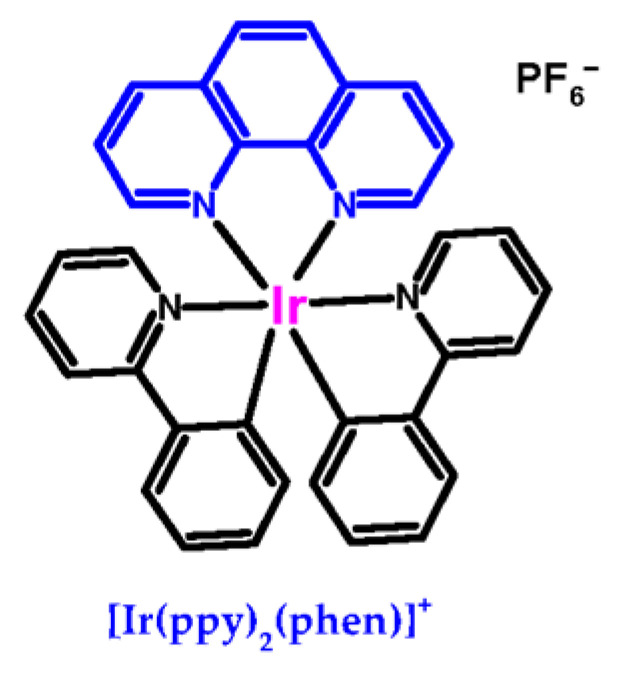	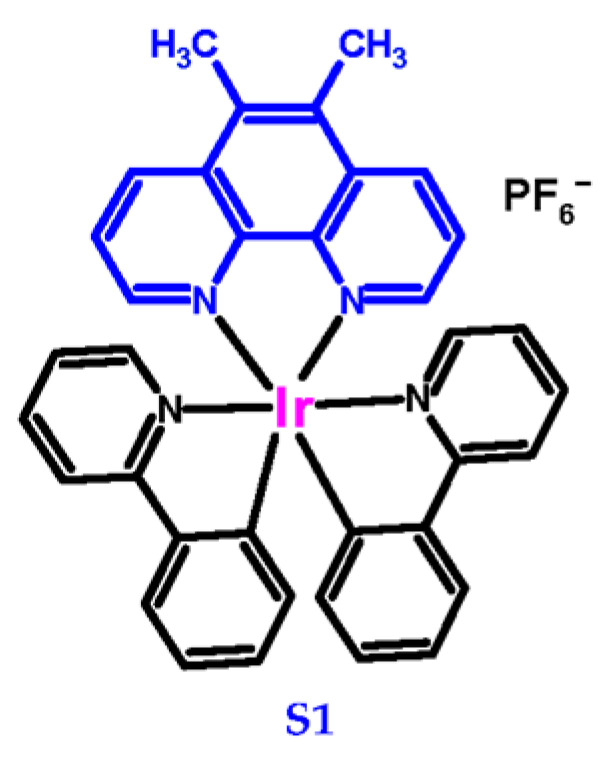			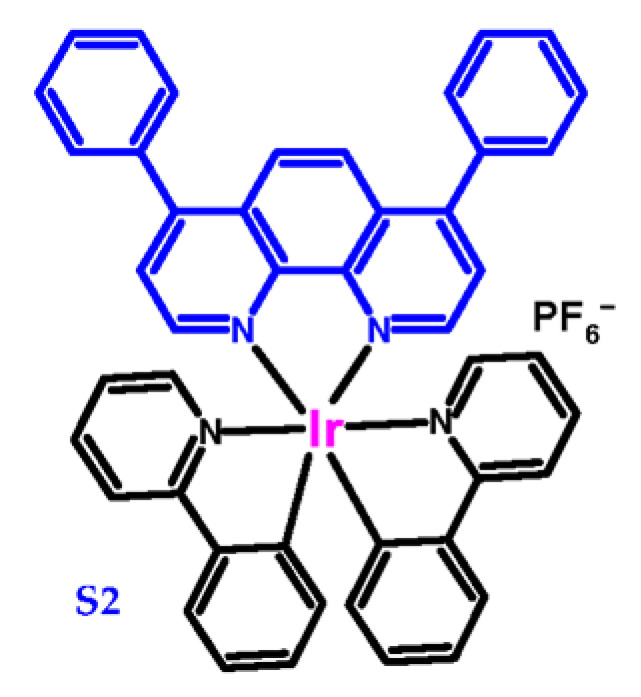		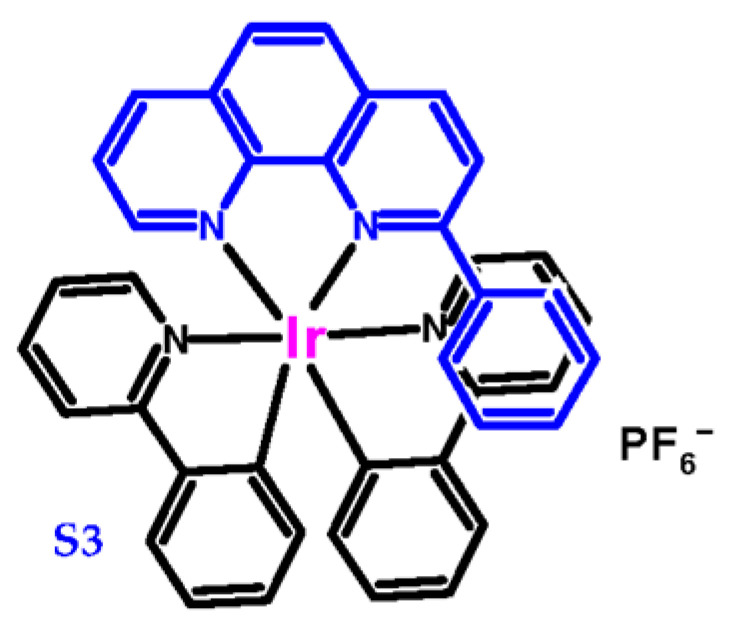			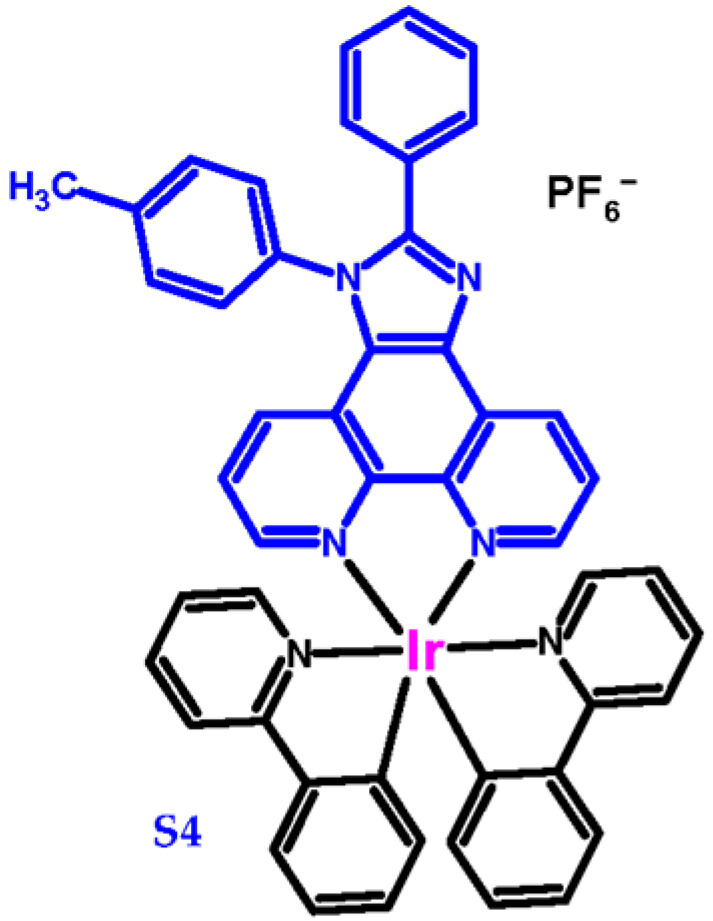	
Ruthenium(II) complexes										
**NE1**	633	2.8	−	−	1500	−	2020	0.53	(0.652, 0.315)	[45]
**NE2**	685	2.6	−	−	2250	−	2100	0.61	(0.662, 0.316)	[45]
**NE3**	660	2.7	−	−	790	−	2200	0.24	(0.690, 0.305)	[45]
**NE4**	662	3.3	−	−	1125	−	1790	0.33	(0.630, 0.308)	[45]
**NE01**	635	2.5	−	−	1965	0.61	3290	0.689	(0.650, 0.312)	[19]
**NE02**	651	2.3	−	−	2395	0.64	3920	0.845	(0.685, 0.310)	[19]
**NE03**	617	2.7	−	−	1615	0.50	3380	0.370	(0.587, 0.359)	[19]
**NE04**	700	3.1	−	−	1300	0.45	2850	1.367	(0.678, 0.336)	[19]
**B1**	635	4.5	539	58	193	0.12	1921	0.141	(0.652, 0.315)	[42]
**B2**	690	3.1	1104	87	742	0.34	2224	0.682	(0.628, 0.309)	[42]
**B01**	661	2.6	667	42	442	0.17	1960	0.69	(0.730, 0.269)	[40]
**B02**	698	2.5	1171	65	731	0.31	2150	0.89	(0.734, 0.265)	[40]
**B03**	645	3.1	486	48	243	0.13	1453	0.59	(0.723, 0.277)	[40]
**F1**	664	2.4	793	36	1066	0.27	3910	1.40	(0.730, 0.269)	[39]
**F2**	695	2.3	528	23	589	0.19	3025	0.93	(0.734, 0.265)	[39]
**F3**	644	2.8	1125	168	878	0.26	3405	1.15	(0.722, 0.277)	[39]
**Bn1**	614	2.6	1794	96	2229	0.65	3310	1.15	(0.677, 0.322)	[41]
**Bn2**	695	2.3	1598	46	1248	0.30	3960	0.93	(0.734, 0.265)	[41]
**Bn3**	706	3.1	875	92	960	0.22	3150	0.510	(0.735, 0.265)	[41]
**E**	702	2.3	94	79	−	−	286	0.62	(0.735, 0.265)	[23]
**ECH_3_^+^**	688	2.6	8.2	27	−	−	309	0.16	(0.735, 0.265)	[23]
**E_2_Ag^+^**	696	2.3	78	12	−	−	322	0.71	(0.734, 0.266)	[23]
**D1**	673	2.9	166	7.1	−	−	196	0.72	(0.732, 0.268)	[44]
**D2**	705	2.6	142	6.3	−	−	151	0.68	(0.735, 0.265)	[44]
**D3**	697	2.7	97	1.4	−	−	342	0.18	(0.735, 0.265)	[44]
**M1**	682	2.7	220	27	−	−	−	−	(0.735, 0.265)	[37]
**M2**	703	3.4	317	44	−	−	−	−	(0.733, 0.266)	[37]
**D**	−	−	−	−	−	−	−	−	−	[37]

## Data Availability

No new data were created or analyzed in this study. Data sharing is not applicable to this article.
